# Supplementary material for: Altered microRNA Transcriptome in Cultured Human Liver Cells upon Infection with Ebola Virus
Source: Int J Mol Sci. 2021 Apr 6;22(7):3792. doi: 10.3390/ijms22073792 (PMC8038836; doi:10.3390/ijms22073792)
Supplement: Supplementary file 1 [file ijms-22-03792-s001.zip › Supplementary_File/D_ KEGG_Analysis_Results/16-30nt_pathway_Reston-24h-Huh7_vs_Control-24h-Huh7_up.mature_mirna_targets/hsa_GeneRatioDotPlot.pdf]

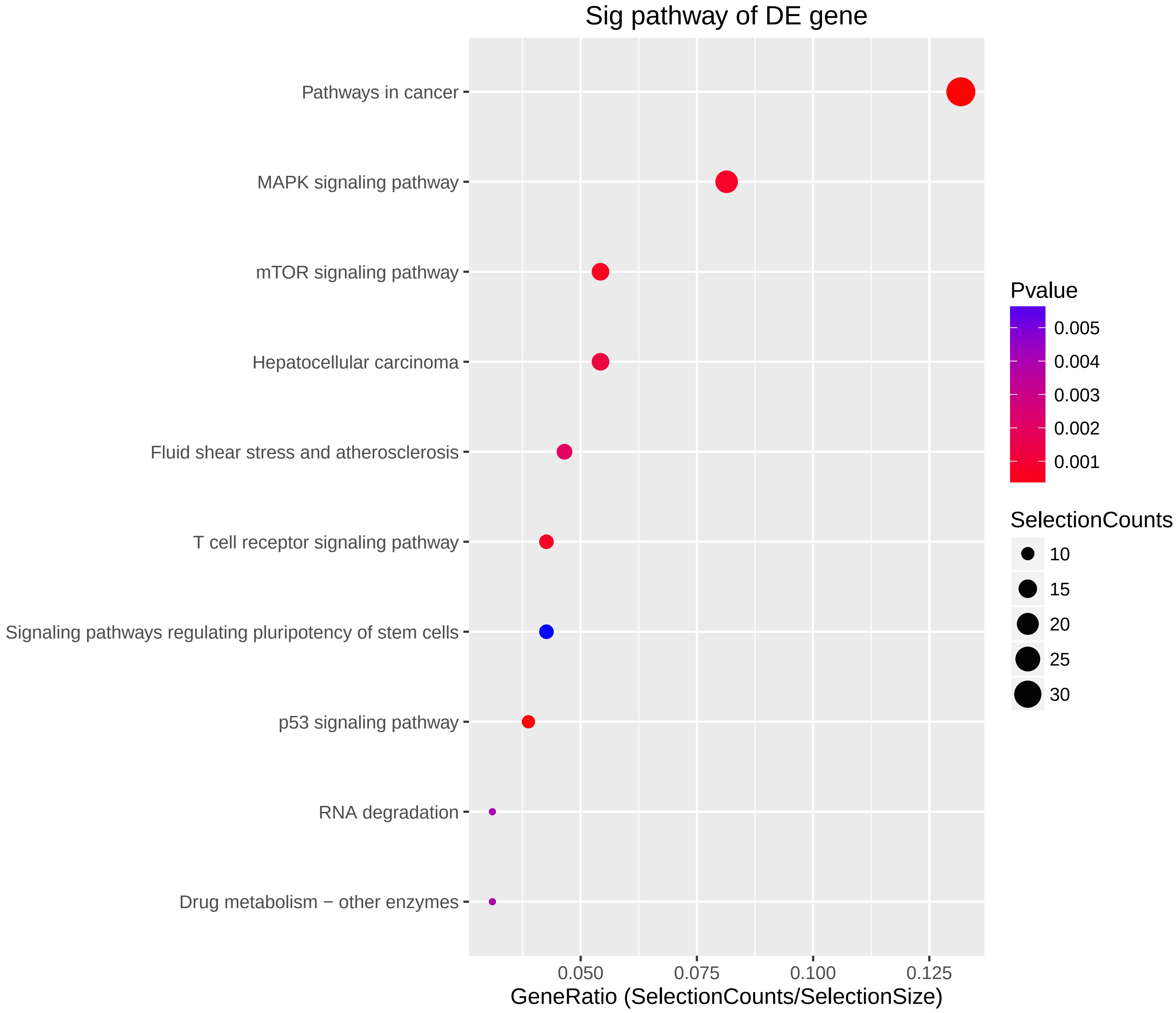

Sig pathway of DE gene
Pathways in cancer
MAPK signaling pathway
mTOR signaling pathway
Hepatocellular carcinoma
Fluid shear stress and atherosclerosis
T cell receptor signaling pathway
Signaling pathways regulating pluripotency of stem cells
p53 signaling pathway
RNA degradation
Drug metabolism − other enzymes
0.050
0.075
0.100
0.125
GeneRatio (SelectionCounts/SelectionSize)
Pvalue
0.005
0.004
0.003
0.002
0.001
SelectionCounts
10
15
20
25
30
